# Comprehensive Biomarker Assessment of Pesticide Exposure and Telomere Attrition in Mexican Children from Agricultural Communities

**DOI:** 10.3390/jox15050141

**Published:** 2025-09-04

**Authors:** Miguel Alfonso Ruiz-Arias, Yael Yvette Bernal-Hernández, Irma Martha Medina-Díaz, José Francisco Herrera-Moreno, Briscia Socorro Barrón-Vivanco, Francisco Alberto Verdín-Betancourt, Cyndia Azucena González-Arias, Eugenia Flores-Alfaro, Kenneth S. Ramos, Patricia Ostrosky-Wegman, Aurora Elizabeth Rojas-García

**Affiliations:** 1 Programa de Doctorado en Ciencias Biológico Agropecuarias en el Área de Ciencias Ambientales, Universidad Autónoma de Nayarit, Xalisco 63000, Nayarit, Mexico; miguelruizarias@hotmail.com; 2 Laboratorio de Contaminación y Toxicología Ambiental, Secretaría de Investigación y Posgrado, Universidad Autónoma de Nayarit, Ciudad de la Cultura s/n, Col. Centro, Tepic 63000, Nayarit, Mexico; irmartha.md@uan.edu.mx (I.M.M.-D.); francisco.herrera@uan.edu.mx (J.F.H.-M.); bbarron@uan.edu.mx (B.S.B.-V.); cyndia.gonzalez@uan.edu.mx (C.A.G.-A.); 3 Secretaría de Ciencia, Humanidades, Tecnología e Innovación (SECIHTI), Padrón de Investigadoras e Investigadores por México, Mexico City 03940, Mexico; 4 Unidad Especializada de Ciencias Ambientales, CENITT. Av. Emilio M. González S/N, Ciudad del Conocimiento, Tepic 63173, Nayarit, Mexico; francisco.verdin@uan.edu.mx; 5 Laboratorio de Investigación en Epidemiología Clínica y Molecular, Facultad de Ciencias Químico-Biológicas, Universidad Autónoma de Guerrero, Chilpancingo 39089, Guerrero, Mexico; eugeniaflores@uagro.mx; 6 Center for Genomic and Precision Medicine, Texas A&M Institute of Biosciences and Technology, Texas Medical Center, Houston, TX 77030, USA; kramos@tamu.edu; 7 Departamento de Medicina Genómica y Toxicología Ambiental, Instituto de Investigaciones Biomédicas, Universidad Nacional Autónoma de México (UNAM), Ciudad Universitaria, Mexico City 05410, Mexico; ostrosky@biomedicas.unam.mx

**Keywords:** pesticide exposure, telomere shortening, biomarkers, cholinesterases, β-glucuronidase

## Abstract

Children are more vulnerable to the adverse effects of pesticides due to physiological factors and behavioral habits. This study aimed to evaluate the impact of pesticide exposure on telomere length (TL) and the enzymatic activity of acetylcholinesterase (AChE), butyrylcholinesterase (BuChE), and β-glucuronidase (β-Glu) in children ages 6 to 12 from an agricultural area in Mexico. A cross-sectional, descriptive, and analytical study was conducted involving 471 children. Blood samples were collected to assess TL through qPCR and enzymatic activity using established protocols. A pesticide exposure index (PEI) was developed incorporating biomarker levels, urinary dialkylphosphates (DAP), and proximity to farmland. No significant differences were observed in AChE activity across communities; however, BuChE activity was significantly higher in agricultural communities, while β-Glu activity varied among communities. Notably, children aged 6 in agricultural areas showed TL values similar to 12-year-old children in the reference community. Adjusted regression models revealed significantly shorter TL in children from agricultural communities and in children with moderate to high PEI. The findings indicate that chronic pesticide exposure was associated with telomere shortening in children, suggesting accelerated biological aging and potential genomic instability during critical developmental periods.

## 1. Introduction

Pesticides are widely used in several sectors, including agriculture, health, urban, veterinary, and domestic settings, to control pests and disease vectors. This extensive use increases environmental pollution [1] and poses health risks for individuals and populations exposed to pesticides. Most research studies on children’s exposure to pesticides have focused on agricultural communities [2,3,4]. However, pesticide contamination is not restricted to agricultural settings, and children in non-agricultural communities may also be exposed [5]. Due to physiological factors, such as lower body mass, differences in detoxification and excretion rates, and certain habits, children can be more vulnerable to the harmful effects of pesticides than adults. Children can be exposed to pesticides through environmental pathways, such as daily intake of contaminated food and contact with polluted soil, water, or air, as well as para-occupationally through contaminated clothing or objects handled by their parents [6,7]. Pesticide exposures have been associated with reproductive and neurodegenerative impairments, as well as the development of some types of cancer, among other chronic effects [8,9,10,11]. The presence of different pesticide chemical classes on children’s hands highlights the complex mixture of chemicals to which they are potentially exposed [12,13]. Pesticides may elicit adverse effects via multiple mechanisms, including oxidative stress, DNA damage, immune alterations, and chronic inflammation [14,15]. Some studies have linked these biological processes to telomere length (TL) [16].

The genetic integrity of the genome is maintained, in part, by the architecture of telomeres. Telomeres are specialized complexes of DNA and proteins located at the ends of chromosomes to protect them from nucleolytic degradation, end-to-end fusion, as well as breakage and inappropriate recombination [17]. Telomeres typically shorten by approximately 50–100 base pairs (bp) with each cell division, reaching a critical length that prevents telomeric DNA replication. TL is a complex trait influenced by multiple factors, including environmental exposures, lifestyle, and genetic background [17,18]. There are previous reports in the literature addressing how maternal stress, sleep hours, obesity, and particulate matter influence TL [19,20,21]. To the best of our knowledge, only one recent study in newborns has reported an association between prenatal exposure to organochlorine (OC) pesticides and TL in umbilical cord blood. The researchers found that higher maternal blood levels of certain OC pesticides were associated with shorter TL in neonates, suggesting that prenatal exposure to these compounds may influence cellular aging from early stages of life [22].

Several studies conducted by our research team demonstrated that in Mexico, particularly in the northwestern region of the country, pesticide use mainly involves organophosphates (OP), followed by pyrethroids (PYR) and carbamates (CB) [23]. Biomonitoring of these pesticide classes in human studies is based on exposure assessment and the use of biomarkers [24,25]. Biomarkers of exposure to OP poisoning are based on the inhibition of acetylcholinesterase (AChE) activity [26], with the degree of severity dictated by the biological condition of the exposed individual, the characteristics of the exposure, and the inherent toxicity of the pesticide [27]. AChE is responsible for chemically hydrolyzing acetylcholine (ACh), removing it from the synaptic cleft, and thus preventing overexcitation of the postsynaptic neuron, which produces clinical manifestations of tremor, vomiting, loss of balance, coma, and death [28]. Butyrylcholinesterase (BuChE), an enzyme structurally related to AChE, is known to hydrolyze compounds such as succinylcholine and bambuterol, which are used as muscle relaxants [29]. Some studies have evaluated the activity of these enzymes as biomarkers of environmental or occupational exposure to pesticides [30,31,32,33]. Other biomarkers have been proposed to assess human exposure to OP. The enzyme β-glucuronidase (β-Glu) is a transferase or hydrolase associated with egasyn, forming a complex in the endoplasmic reticulum membrane. Egasyn is a member of the serine esterase family expressed primarily in the liver. Upon entry, OP binds strongly to egasyn, releasing β-Glu into the bloodstream. The Egasyn-OP complex remains within the hepatocytes while β-Glu is secreted to the plasma, leading to increased enzymatic activity. As such, increased plasma activity of β-Glu has been used as a biomarker of OP exposure [34]. However, there is a scarcity of studies assessing changes in blood β-Glu activity in acute OP poisoning in humans [24,35].

One of the most widely accepted biomarkers for measuring internal dosing following OP exposure is the presence of dialkylphosphates (DAP) in urine. DAP metabolites of interest include dimethylphosphate (DMP), diethylphosphate (DEP), dimethylthiophosphate (DMTP), diethylthiophosphate (DETP), dimethyldithiophosphate (DMDTP), and diethyldithiophosphate (DEDTP). Measurement of these metabolites constitutes a fundamental tool for assessing exposure [36,37]. Since the determination of pesticide exposure through urinary metabolites or enzyme activity measurements can lead to high experimental and logistical costs, researchers have also relied on exposure indices or proxy indicators to estimate levels of pesticide exposure [38]. The construction of these indices compares exposure scores using information from questionnaires or may consider the distance between households and agricultural fields [38,39]. The use of these indices for epidemiological analysis helps to generate alternative strategies to assess the health risks associated with pesticide exposure.

The literature for exposure biomarkers such as AChE and BuChE in children is extensive [40,41,42,43,44]; however, data on other biomarkers, such as β-Glu activity, in children worldwide, as well as the assessment of DAP in Mexican children, are limited [45,46,47,48,49,50]. The present study aimed to evaluate the effect of pesticide exposure, measured through DAP metabolites in urine, residues on children’s hands, and the pesticide exposure index (PEI), on TL and the enzymatic activity of AChE, BuChE, and β-Glu in children from an agricultural area in Mexico.

## 2. Methodology

### 2.1. Study Population

A cross-sectional, descriptive, and analytical study was conducted on 471 children aged 6 to 12 years from three different communities. Community A has a high agricultural production (49,343.5 ha) as well as intensive pesticide use. Its main crops included beans, rice, corn, tobacco, and mango [51], while the most frequently used pesticides include N-(phosphonomethyl) glycine (N-PMG), PYR, bipyridyls, and neonicotinoids (NEO). In Community B, the main crops were beans, rice, and tobacco [51], while the most frequently used pesticides were PYR, N-PGM, and OP pesticides [23,52]. Community C, although distant from agricultural fields, shared similar sociodemographic characteristics with Communities A and B. Its main economic activities included retail trade, food and beverage services, the manufacturing industry, and healthcare services [53].

Parents of students from participating schools were invited to attend information sessions, during which the objectives of the study were explained in detail. Subsequently, information leaflets and informed consent forms were distributed to all students of each participating institution. Only those students whose parents voluntarily signed the consent forms were considered eligible for inclusion in the study. In addition, on the day of the sample collection, the participating children gave their written consent, indicating their willingness to participate in the research.

The participation rates were as follows: 60.2% in Community A, 61.7% in Community B, and 60.5% in Community C, thus producing a representative sample of each study population. The research protocol was reviewed and approved by the Bioethics Committee of the State of Nayarit (CEBN/01/2022).

In addition, the study was completed in collaboration with professionals who participated in the collection of biological data and samples, as well as analytical processing. Anthropometric measurements, including height, weight, body mass index (BMI), and other relevant parameters, were performed by a qualified nutritionist.

Blood samples were collected from each participant using BD vacutainer^®^ plastic tubes to evaluate TL, AChE, BuChE, and β-Glu. Samples were obtained after an overnight fast of 8 to 12 h. Hemoglobin (Hb) analysis was performed using a Sysmex XN-L (XN-550, Kobe, Japan) hematology analyzer. Whole blood aliquots were separated for DNA extraction. The tubes were centrifuged at 3500 rpm for 10 min to obtain serum and plasma. The samples were stored at −80 °C until analysis. Urine samples were collected in 100 mL sterile containers. Parents or guardians were asked to assist in collecting the first-morning urine samples from the participating children. The samples were collected by the research team at the schools, labeled, and transported to the laboratory, where they were divided into 15 mL aliquots and stored at −80 °C.

### 2.2. Proximity of School to Agricultural Fields

The distance between participating schools and nearby agricultural fields was measured for each community in the study population. Google Earth^®^ pro version 10.88.0.3 was used as a geolocation tool to measure the distance between two points with respect to each location to be measured.

In Community A, the participating elementary school was located 431.33 m from the nearest crop fields. Community B has two elementary schools, with an average distance of 309.67 m to nearby agricultural fields (388.10 m for school 1 and 231.25 m for school 2). Community C was situated well beyond agricultural operations, with its participating school located 2383.25 m from the nearest fields. Across all three communities, children have witnessed pesticide applications by aircraft used for vector control, highlighting their potential environmental exposures [54].

### 2.3. Pesticide Exposure Assessment

To assess pesticide exposure, 30 children from each community were randomly selected for handwashing samples. Handwashing samples were collected using a procedure adapted from van Wendel de Joode et al. [55]. Briefly, each child was asked to immerse and rub their hands inside a beaker (2 L) containing 0.6 L of purified water. Handwashing samples from all children in the same community were pooled, as previously reported by Ruiz-Arias et al. [13].

Pesticide analysis, which covers both handwashing samples and the quantification of DAP metabolites, was completed to evaluate the presence of higher levels of pesticide exposure in test communities.

### 2.4. DNA Extraction

The SpeeDNA Isolation Kit (SPDNAI) with catalog number MB6918-1 (ScienCell™ Research Laboratories, San Diego, CA, USA) was used according to the manufacturer’s instructions. Briefly, 100 µL of whole blood was used for DNA extraction. Proteinase K was added to degrade proteins by hydrolyzing peptide bonds and to eliminate nucleases that could degrade genetic material. Separation was performed using a silica membrane spin column-based purification method. The eluate was obtained by adding 80 µL of the elution buffer supplied with the kit. DNA purity and concentration were determined using the NanoDrop 2000c equipment (Thermo Fisher Scientific, Waltham, MA, USA). The geometric mean (GM) purity of the DNA was 2.0 (95% CI: 1.99, 2.03).

### 2.5. Telomere Length

TL was measured using the Absolute Human Telomere Length and Mitochondrial DNA Copy Number Dual Quantification qPCR Assay Kit (AHDQ), catalog number #8958 (ScienCell™ Research Laboratories, San Diego, CA, USA). Real-time PCR assays were performed using StepOne™ ver 2.1 Applied Biosystems software (Foster City, CA, USA). The experiment was conducted using a Comparative Quantitation CT (ΔΔCT) method with SYBR^®^ Green reagents, including a melting curve analysis. The PCR was performed at standard speed (∼2 h to complete one run), and the ROX passive reference dye option was disabled. Telomere primer set and single copy reference primer set (SCR) were reconstituted by adding 200 μL of nuclease-free H_2_O. The PCR reaction mixture contained 1 μL of genomic DNA template (5 ng/μL) or reference genomic DNA sample, 2 μL of primer stock (telomere or SCR), 10 μL of 2X GoldenNStart TaqGreen qPCR master mix, and 7 μL of nuclease-free H_2_O. Individual reactions were set up for telomere and SCR primers. PCR reaction conditions consisted of the following: 95 °C for 10 min; then 32 cycles at 95 °C for 20 s, 52 °C for 20 s, and 72 °C for 45 s, with data acquisition. A final melting and retention curve analysis was included (20 °C for 2 min). The TL and SCR quantifications were performed according to the manufacturer’s protocol.

### 2.6. Acetylcholinesterase Activity

AChE activity was determined using the method described by Ellman et al. [56]. Erythrocytes were lysed with 1 mL of a 1:100 dilution of the blood sample using triton X-100 nonionic detergent. The reaction mixture contained 0.5 mL of a 1:100 dilution of phosphate buffer. The reaction mixture contained 0.5 mL of the 1:100 dilution, 1 mL of phosphate buffer (0.1 M, pH 7.4), 0.05 mL of 5,5′-Dithiobis-(2-nitrobenzoic acid) (DTNB) (10 mM), and 0.005 mL of ethopropazine (6 mM). The mixture was incubated at 37 °C for 10 min, and then 0.025 mL of acetylthiocholine iodide (28.3 mM) was added. The change in absorbance at 436 nm was monitored immediately every minute for 3 min. AChE activity was corrected for Hb content and expressed as U/g Hb.

### 2.7. Butyrylcholinesterase Activity

The BuChE activity was determined according to the method described by Ellman et al. [56]. A reaction mixture contained 3 mL of phosphate buffer (0.1 M at pH 7.4), 0.100 mL of DTNB (10 mM), and 0.010 mL of serum. The mixture was gently mixed and incubated at 37 °C for 10 min. Then, 0.050 mL of S-butyrylthiocholine iodide (63.2 mM) was added. The change in absorbance was monitored at 405 nm for 4 min, every minute. The measurement of enzyme activity was reported in U/L.

### 2.8. β-Glucuronidase Activity

β-Glu activity was determined by the method of Stahl and Fishman [57] and Hernández et al. [58], with modifications by Ruíz-Arias et al. [59]. The reaction mixture contained 200 μL of plasma, 200 μL of sodium acetate buffer (1 M), 200 μL of phenolphthalein glucuronide (0.03 M), and 400 μL of bi-distilled water. The reaction was incubated for 4 h at 38 °C. After incubation, 5 mL of 0.2 M glycine/0.2% SDS solution was added to alkalize the medium (pH 10.5). The color change to a pinkish hue was observed. The samples were then centrifuged at 3000 rpm for 10 min, and the absorbance was measured at 540 nm. The color intensity is directly proportional to the enzyme activity. The reaction product (phenolphthalein) is stable at room temperature and can be stored at 4 °C for more than 12 months. The measurement of enzyme activity was reported in U/dL.

### 2.9. Dialkylphosphates (DAP)

Groups of 14–17 urine samples were pooled for each community (A, B, and C). Six main metabolites of OP were measured: DMP, DMTP, DMDTP, DEP, DETP, and DEDTP. Quantification was performed by using a Gas Chromatography–Mass Selective Detector (GC-MSD), following the method described by Valcke et al. [60], with modifications reported by Ramírez-Jiménez et al. [50]. Metabolite concentrations were corrected for creatinine using a validated kit (Jaffe Kinetics) from Valtek diagnostics, Las Condes, Santiago de Chile, Chile (cod. 8001214) following the manufacturer’s instructions. Data are presented in nmol/g creatinine. These results were previously reported in Aguilar-Bañuelos et al. [61] and Ruiz-Arias et al. [13] as medians and 25th and 75th percentiles.

### 2.10. Pesticide Exposure Index (PEI)

To assess the degree of exposure to pesticides, we constructed a PEI as an indicator of exposure among children in Communities A, B, and C. The PEI was calculated by summing the variables of enzymatic activities and environmental exposures as described above. Variables were organized into two levels according to the GM of the biomarkers used and were assigned a value of 0 or 1. For AChE, GM = 27.02 U/g Hb, and BuChE 4730.42 U/L, values were assigned as 0 ≥ GM and 1 < GM. β-Glu (GM = 7.94 U/dL), DAP (GM = 2385.8 nmol/g creat), and pesticides on hands (GM = 14.12 nmol/L) were assigned as 0 < GM and 1 ≥ GM. The distance of schools to agricultural fields in Communities A, B, and C was assigned as 0 ≥ 500 m and 1 < 500 m. The PEI was calculated as follows:

PEI = AChE + BuChE + β-Glu+ pesticides on hands + DAP + distance of schools to agricultural areas

PEI ranged from 0 to 6, with 0 representing the lowest exposure and 6 the highest. Children were grouped into three exposure levels on their PEI: low exposure of 0–2 (*n* = 141), moderate exposure of 3 (*n* = 165), and high exposure of 4–6 (*n* = 165).

### 2.11. Statistical Analysis

Descriptive analyses of the study population and exposure biomarkers were conducted using GM and 95% confidence intervals (95% CI). The Mann–Whitney U test and Kruskal–Wallis test, followed by Dunn’s post hoc test, were used to assess statistical differences (*p* < 0.05). Spearman correlation analyses were performed to examine the relationships between exposure biomarkers. Generalized linear regression models (GLM) were used to evaluate the association between biomarkers and pesticide exposure by community of residence. Results are reported as linear regression coefficients (β) with 95% CIs. All models were adjusted for age- and sex, a potential confounder. BMI categories were defined using sex-specific BMI-for-age percentiles based on U.S. Centers for Disease Control and Prevention (CDC) [62] guidelines. Data were analyzed using Stata version 14 (StataCorp, College Station, TX, USA), and graphs were generated with GraphPad Prism version 9.0 (San Diego, CA, USA).

## 3. Results

### 3.1. Study Population Description

Table 1 presents the characteristics of the study population, comprising 471 children from Communities A (38.85%), B (32.48%), and C (28.66%). Boys and girls participated in equal proportions across the three communities. Children in Communities A and B showed marginally higher BMI values compared to the reference population (Community C), and more than 47% of the children from agricultural communities were overweight and obese.

As recently reported by Ruiz-Arias et al. [13], participants in this study had pesticide residues from different chemical classes on their hands, including benzimidazole (BZ) (carbendazim, thiabendazole), CB (propamocarb), N-PMG (AMPA, a glyphosate metabolite), NEO (imidacloprid, thiamethoxam), OC (p,p′-dichlorodiphenyldichloroethane-DDD, p,p′-dichlorodiphenyldichloroethylene-DDE, p,p′-dichlorodiphenyltrichloroethane-DDT), OP (diazinon, chlorpyrifos, malathion), PYR (permethrin, bifenthrin), and others. The GM of the total pesticide concentration was higher in Communities A (49.77 nmol/L) and B (45.1 nmol/L) than in Community C (1.14 nmol/L). These data were used in the construction of the PEI.

### 3.2. Organophosphates Metabolites (DAP)

Table 2 shows the descriptive analysis of the concentration of OP pesticide metabolites (DAP) in the urine of the studied population. The most frequently detected DAP metabolite was DETP (100% of the samples). The DEDTP and DMP metabolites were only detected in participants residing in Community A. In addition, children from the agricultural communities had significantly higher summed levels of total DETP, DMP, and DAP compared to Community C.

### 3.3. Telomere Length and Enzymatic Activities

**Figure 1:**
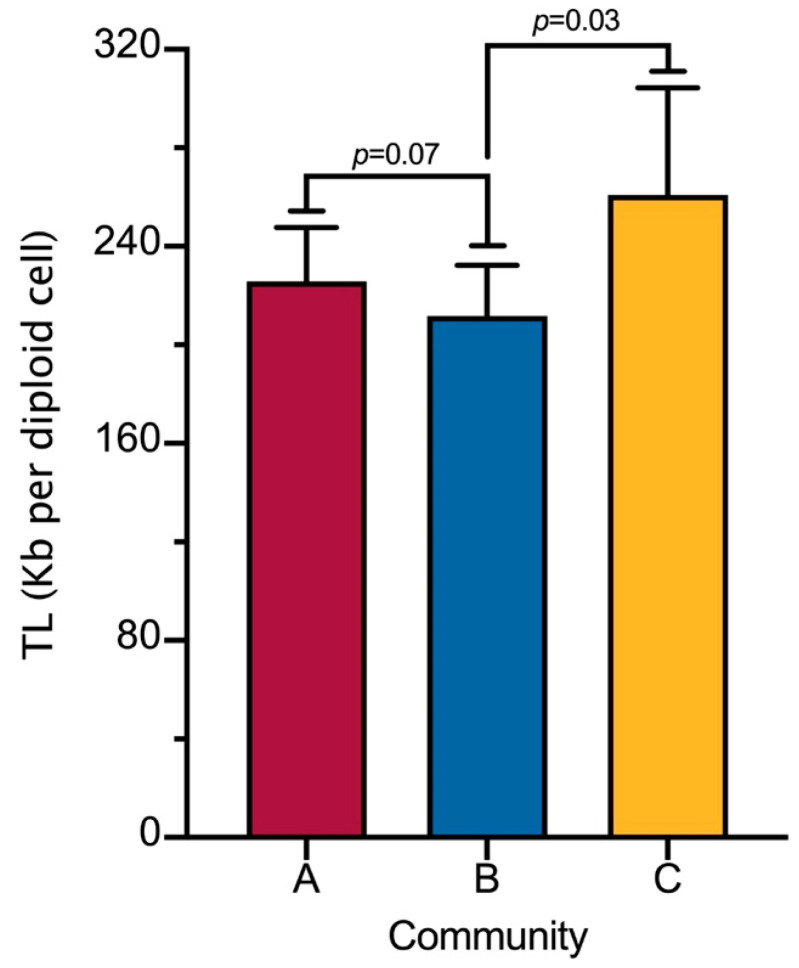
Telomere length (TL) by study community. Data show the geometric mean of the activities and 95% confidence intervals. Statistical analysis was performed with the Kruskal–Wallis test followed by Dunn’s post hoc test. *p* < 0.05 was considered statistically significant.

Figure 1 shows the difference in TL between communities. The GM of the communities in TL per diploid cell was 225.8 kb (95% CI: 205.8, 247.7) for community A, 211.8 kb (95% CI: 193.1, 232.4) for Community B, and 260.8 kb (95% CI: 223.4, 304.436) for Community C. Significantly shorter TL was observed in Community B with respect to Community C.

**Figure 2:**
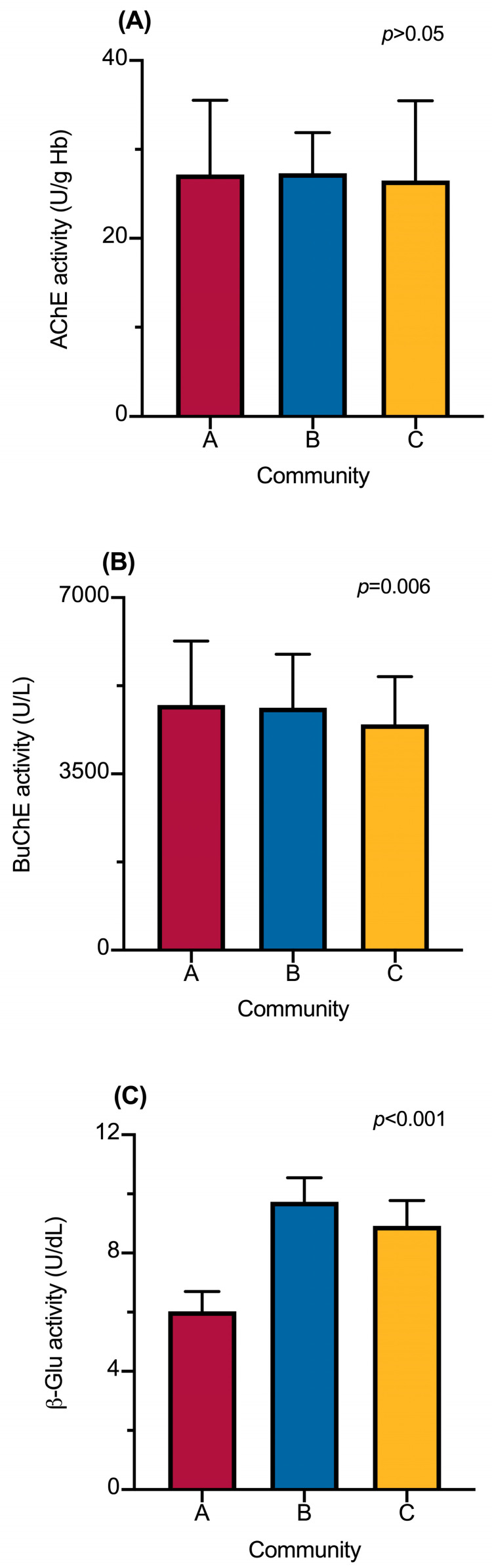
Enzymatic activities in the study population. (**A**) Acetylcholinesterase (AChE), (**B**) Butyrylcholinesterase (BuChE), and (**C**) β-Glucuronidase (β-Glu) activities in the study population. Data show the geometric mean of the activities and 95% CI. Statistical analysis was performed with the Kruskal–Wallis test followed by Dunn’s post hoc test. *p* < 0.05 was considered statistically significant.

Regarding the enzymatic activity of AChE, BuChE, and β-Glu in the study population, no significant differences were observed in the activity of AChE among the communities (*p* > 0.05) (Figure 2A). However, BuChE was significantly higher in the agricultural communities (community A: 4869.1 U/L, and Community B: 4811.7 U/L) compared to the reference community (4482.5 U/L) (Figure 2B). The activity of β-Glu was higher in Community B (9.7 U/dL) but lower in Community A (6.0 U/dL) compared to the reference community (8.9 U/dL) (Figure 2C).

### 3.4. Correlation Between Telomere Length and Age Among the Study Population

**Figure 3:**
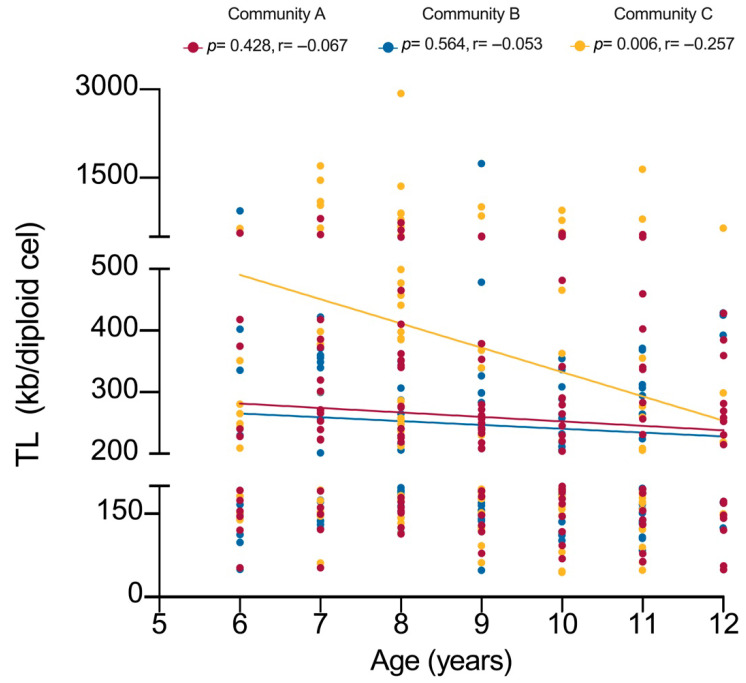
Correlation between telomere length (TL) and age, stratified by community. Correlation coefficients were calculated using Spearman’s rank correlation. *p* < 0.05 was considered statistically significant.

Figure 3 shows the correlation between TL according to community and age of the children. As expected, a significant negative correlation between TL and age was observed; however, it was only observed in children from the reference community. In the case of the children from the agricultural communities (A and B), those aged 6 years presented TL values similar to those observed in the 12-year-old children from the reference community (C).

### 3.5. Telomere Length and BMI Categories

**Figure 4:**
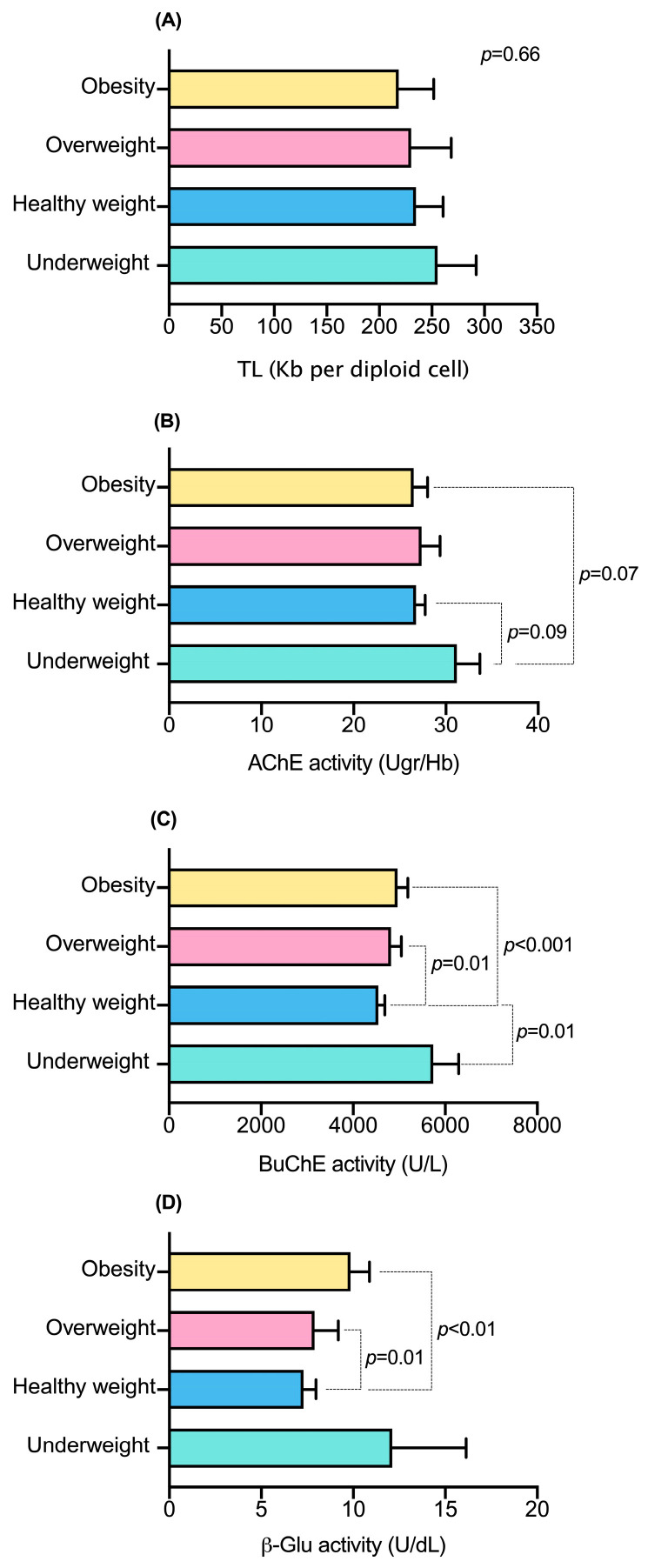
Telomere length (TL) and enzymatic activities according to BMI categories in the study population. (**A**) telomere length (TL, Kb per diploid cell), (**B**) acetylcholinesterase (AChE, U/g Hb), (**C**) butyrylcholinesterase (BuChE, U/L), and (**D**) β-glucuronidase (β-Glu, U/dL). Data show the geometric mean and 95% confidence interval. Statistical analysis was performed using the Kruskal–Wallis test, followed by Dunn’s post hoc test. *p* < 0.05 was considered statistically significant.

Figure 4 shows TL, AChE, BuChE, and β-Glu according to BMI categories. The data suggest that BMI is not related to TL in the study population, in contrast to the activity of BuChE and β-Glu, where overweight or obese children have higher activity of these enzymes compared to healthy-weight children.

### 3.6. PEI in the Study Population

The low exposure group, stratified by PEI, consisted of more than 80% of children from Community C (reference community). In contrast, the moderate and high exposure groups consisted mainly of children from the agricultural communities (Communities A and B). No significant differences were observed in the sex distribution according to the group studied. Although no significant differences in BMI were found between PEI categories, at least 45% of the children in the moderate and high exposure groups were classified as obese or overweight. We acknowledge that, as a cross-sectional study, our design captures a single time point and does not allow us to infer causality or assess the exact duration or frequency of pesticide exposure. Nevertheless, the detection of pesticide residues on participants’ hands and the observed proportions of overweight and obesity in children with moderate to high exposure to pesticides provide valuable insights into the growing body of evidence on environmental determinants of pediatric populations exposed to pesticides. According to PEI categories, AChE and BuChE enzymatic activities decreased as PEI increased, whereas β-Glu activity, OP residues on hands, and DAP metabolites increased according to the increase in exposure.

### 3.7. Effect of Pesticide Exposure, Assessed by Community of Residence and PEI, on Telomere Length Reduction

Unadjusted and adjusted GLMs were used to assess the effect of communities (A and B) and PEI on TL reduction (measured in kb per diploid cell). After adjusting for age and sex, we found significant TL reductions of 114.7 kb (Community A) and 129.2 kb (Community B) compared to the reference Community C. Similarly, moderate and high PEI levels showed effects corresponding to TL reductions of 82.8 kb and 85.8 kb, respectively, relative to low exposure (age- and sex-adjusted models) (Table 3).

## 4. Discussion

### 4.1. Pesticide Exposure Biomarkers

In recent years, the detection of pesticide residues across the environment has increased significantly. Elevated levels of these compounds have been found in soil, water, and plant and animal species in various regions of Mexico. Moreover, studies have identified adverse effects on human health, specifically among children [63]. Previous research has documented high pesticide usage [23,64,65] and the presence of OP compounds in environmental matrices in Nayarit, Mexico [52], as well as OP pesticide metabolites and at least 18 different pesticides on children’s hands in our study area [13]. Rural households tend to have higher concentrations of pesticide residues compared to urban households, primarily due to the greater variety of pesticides used in agricultural settings [66].

The results showing pesticide residues on children’s hands indicate significant exposure to chemical families, including BZ, CB, N-PMG, NEO, OC, OP, PYR, and others. This diversity of residues points to the continuous and complex exposure to pesticide mixtures in agricultural areas. Furthermore, direct exposure through the hands represents a critical route, particularly in children. According to a model by Li et al. [67], a 25-year-old adult and a 10-year-old child ingest 6.2 mg and 20.9 mg of dust daily through hand-to-mouth contact, respectively. Research has indicated a positive relationship between the levels of chemicals found on hands and their concentrations in human blood [68,69].

It is important to emphasize that the three OP pesticides most frequently detected in children (diazinon, chlorpyriphos, and malathion) are biotransformed into DAP metabolites [70], which were used in the present study as biomarkers of internal exposure to OP.

Studies have documented that both OP and CB inhibit AChE and BuChE activities in agricultural workers and environmentally exposed populations. A longitudinal study with pesticide handlers from the Washington State Cholinesterase Monitoring Program (2006–2011) demonstrated a significant seasonal decline in BuChE activity, with the greatest reductions seen among those who mixed or applied multiple OP/CB formulations [31]. Likewise, it has been shown that chronic exposures to OP in agricultural workers are associated with a significant decrease in AChE and neurotoxic effects [71].

Regarding DAP concentrations in urine, the detection and concentration of these metabolites in our study population provide strong evidence of exposure to OP pesticides. The fact that DETP was detected in 100% of the urine samples indicates widespread and consistent exposure to OP pesticides across the entire study population, highlighting a potential baseline environmental contamination or residual exposure even in non-agricultural areas. Most notably, children from agricultural communities (A and B) exhibited significantly higher concentrations of multiple DAP metabolites, including DEP, DETP, DMTP, and DMDTP, compared to those in the reference community (C). The higher summed concentrations of total DAPs reinforce the conclusion that living in, or near, areas of intensive pesticide application substantially increase children’s internal dose of OP pesticides. These findings are consistent with previous research demonstrating elevated urinary DAP levels in children living in agricultural areas or in homes where pesticides are frequently used [72,73]. This high internal exposure is of concern in children, who may be more vulnerable to the neurotoxic effects of OP pesticides due to their developing nervous system [74,75].

Data published previously indicate that DAP concentrations ranging from 1.8 µg/g creatinine (low exposure period) to 2.2 µg/g creatinine (high exposure period) are found in children from south-eastern Spain [76]. In the Center for Health Assessment of Mothers and Children of Salinas (CHAMACOS) cohort, DAP metabolites were measured in both maternal and child urine to characterize OP exposure over time. Specifically, prenatal urine samples were collected from mothers and postnatal samples from children between 6 months and 5 years of age. The GM of total DAP concentration in maternal urine was 109.0 nmol/L (95% CI: 99.4, 119.6), while in children, it was 77.5 nmol/L (65.4, 91.9) at 3.5 years and 92.6 nmol/L (78.6, 109.0) at 5 years. Diethyl metabolites had a GM of 17.7 nmol/L in mothers, with children showing 7.0 nmol/L at 3.5 years and 7.2 nmol/L at 5 years; dimethyl metabolites had a GM of 76.8 nmol/L in mothers, with children showing 62.5 nmol/L at 3.5 years and 72.4 nmol/L at 5 years. These findings indicate chronic, pervasive OP exposure across all stages studied and are in keeping with the major findings of our study [77]. In Mexico, to the best of our knowledge, there are only two published studies that have evaluated DAP concentrations in children in a study population of children and adolescents (6 to 14 years old) from an agricultural area in the state of San Luis Potosí during periods of lower and higher pesticide application [50,78]. The authors mention that during the high exposure period, the sum of the DEP metabolites concentration was 57 nmol/g creatinine, while the sum of DMP was 158 nmol/g creatinine and the total DAP concentration was 216 nmol/g creatinine [50]. Also, the data reported by Ramírez-Jiménez et al. [50] and Yáñez-Estrada et al. [78] were lower than the concentrations found in our study related to DAP metabolites, which are within the range reported in workers occupationally exposed to pesticides (127.7–2186.4 µg/g creatinine) [79].

In addition, the analysis of the PEI in this study integrates several exposure biomarkers (e.g., OP metabolites in urine, residues on hands) and effect biomarkers (e.g., enzymatic activities) and reveals clear trends related to geographical location, biological outcomes, and certain demographic variables, even in the absence of statistically significant differences for some parameters.

### 4.2. Pesticide Effects on Telomere Length

On the other hand, TL is a recognized marker of biological aging and genomic instability [80]. Studies have suggested that environmental exposures to toxic substances, including pesticides, may influence the dynamics of telomere shortening or lengthening, reflecting an imbalance in DNA damage and repair processes [16,81,82,83,84].

In the current study, children from agricultural communities showed significantly different TL compared to the reference group, suggesting a biological effect potentially attributable to environmental and lifestyle exposures, including pesticides.

The relationship between pesticide exposure and TL represents an emerging area in environmental and toxicological research. Available evidence suggests that pesticides, particularly OP and OC, may act as accelerators of cellular aging, which is especially relevant for vulnerable populations such as children or agricultural workers exposed from an early age [22,84,85]. Data in the literature indicate that higher levels of pesticide exposure were associated with shorter telomeres [86].

Several studies have identified that environmental exposure to pesticides can induce oxidative stress, inflammation, and DNA damage, key mechanisms involved in the acceleration of telomere shortening [15,61,87,88,89,90,91]. Overall, telomeres are very sensitive to oxidative stress damage due to the high guanine content in telomeric sequences [92]. Furthermore, pesticides can cause alterations in DNA methylation patterns [93] and influence telomerase activity [94].

Studies conducted in Brazilian tobacco farmers found a significant decrease in TL in the pesticide-exposed group compared to the unexposed group [95,96]. This is consistent with our results, since generalized linear models show a shortening of TL in agricultural communities compared to the reference group.

### 4.3. Enzymatic Activities of Cholinesterases and β-Glucuronidase Associated with Pesticide Exposure

In addition, the evaluation of effect and exposure biomarkers, such as AChE, BuChE, and β-Glu, provides valuable information on potential physiological alterations associated with pesticide exposures. In this study, no significant differences were observed in AChE activity between the study groups, which may be due to various factors, including interindividual variability, the type and duration of exposure, or the existence of physiological compensatory mechanisms. Although AChE is a classic biomarker of inhibition by OP and CB, what our research team has found is that it is a less sensitive biomarker compared to BuChE [97].

There are reports of AChE activity in children from agricultural populations [40,41,98,99,100,101,102,103]. Research has found that living near crop fields is associated with lower AChE activity in children residing less than 275 m from farmland [104]. However, our results did not align with these findings, even though we studied children at similar distances (greater than 500 m). Previous research by Díaz-Romo and Salinas-Álvarez [64] reported AChE activity in children from Community A, where Huichol day laborer children exhibited AChE inhibition during harvest periods, dropping from a baseline of 28.91 U/g of Hb to 27.5 U/g of Hb during harvest. In contrast, mestizo children showed a baseline activity of 26.61 U/g of Hb, which increased to 28.02 U/g of Hb during the same period.

On the other hand, BuChE activity was significantly higher in the agricultural communities compared to the reference group, which could be interpreted as an adaptive response of the organism to prolonged pesticide exposure, specifically to OP. A significant increase in BuChE activity was observed in agricultural populations, alongside a higher prevalence of obesity and overweight individuals. Those with hyperlipidemia and obesity, particularly those with abdominal obesity, exhibited elevated levels of the BuChE enzyme compared to individuals of normal weight [105]. In a 2018 study in a Mexican child population, Ramírez-Jiménez et al. [106] reported a noticeable increase in BuChE activity among overweight and obese children.

Our study is the first to highlight the potential utility of measurements of β-Glu enzymatic activity in children exposed to pesticides. Previous studies have evaluated the increase of β-Glu activity in adults who have been occupationally exposed to those xenobiotics [34,107,108,109]. Our data show that β-Glu, a lysosomal enzyme associated with inflammation and tissue damage, showed higher activity in Community B but lower activity in Community A compared to the reference group. These results may reflect differences in the types of pesticides used, local agricultural practices, or other factors. These contrasting results underscore that pesticide exposure does not produce uniform biological effects across populations. Therefore, relying on a single biomarker could be insufficient. Instead, assessing multiple biomarkers—including β-Glu activity, alongside others—provides a more comprehensive picture of exposure effects, capturing variations in exposure route, intensity, and chronicity.

Recently, compounds from the azole families (pyrazoles, imidazoles, thiazoles, triazoles, oxadiazoles, thiadiazoles, and tetrazoles) have been described as potent inhibitors of β-Glu activity [110]. This aligns with a recent report from our team documenting complex mixtures of pesticides in the study area [13,23].

Finally, the study population of this work is undoubtedly exposed to OP pesticides, and OP has been linked to obesity through mechanisms that include neurofunctional impairment, sleep disturbances, gastrointestinal dysfunction, and metabolic disorders [111,112]. In this study, a correlation between BMI and the activities of BuChE and β-Glu, as well as the presence of OP on children’s hands and the distance between schools and agricultural fields, was observed, which might implicate a risk of obesity in children, generating hormonal imbalances, chronic inflammation, and other chronic diseases [59,107,113].

This study has limitations. The cross-sectional design and relatively small sample size restrict the ability to establish causal relationships and limit the generalizability of findings to larger populations. Moreover, the pooling of handwashing and urine samples at the community level masks interindividual variability in both internal and external pesticide exposure. Nevertheless, the use of pooled samples represents a valid and logistically justified methodological approach, widely recognized for its ability to obtain representative data from population groups by combining individual subsamples. Despite these limitations, the study has notable strengths, including the participation of children from multiple communities with comparable sociodemographic profiles and the inclusion of key covariates, such as BMI, in the multivariate analyses. Notably, this research is among the very few studies to simultaneously investigate the interplay between pesticide exposure, biochemical biomarkers, and TL in a pediatric population, highlighting its novelty and contribution to the field.

## 5. Conclusions

In conclusion, the elevated levels of OP metabolites found in children, along with alterations in key enzyme activities, provide compelling evidence of chronic exposure with potentially severe health consequences. The detection of pesticide residues even within household environments highlights the pervasive nature of this concern. Given that the concentrations of DAP metabolites in these children exceed those reported in agricultural workers globally, it is imperative to implement stricter regulations on OP pesticide use to protect vulnerable populations such as children. The findings of this study provide evidence that telomere shortening is associated with pesticide exposure in a Mexican child population, particularly among those living in agricultural communities. This suggests that chronic environmental exposure to pesticides may accelerate biological aging processes and contribute to genomic instability during critical periods of growth and development. Given the potential long-term health implications, including increased susceptibility to chronic diseases, these findings underscore the urgent need for public health interventions, environmental monitoring, and protective regulations aimed at reducing pesticide exposure in vulnerable populations. Further longitudinal studies are needed to clarify the causal mechanisms and to assess the reversibility or progression of telomere attrition over time.

## Figures and Tables

**Table 1:** Characteristics of study population.

Characteristics	Community A	Community B	Community C	*p* Value
Total [*n* (%)]	183 (38.8)	153 (32.4)	135 (28.6)	
Sex				0.63 ^a^
Male [*n* (%)]	87 (47.5)	77 (50.3)	68 (50.3)	
Female [*n* (%)]	96 (52.4)	76 (49.6)	67 (49.6)	
Age [years (95% CI)]	8.6 (8.4, 8.9)	8.63 (8.3, 8.9)	8.73 (8.4, 9.0)	0.60 ^b^
BMI [Kg/m^2^ (95% CI)]	19.3 (18.6, 20.0)	19.41 (18.6, 20.1)	18.27 (17.6, 18.9)	0.07 ^b^
Underweight [*n* (%)]	7 (4.3)	2 (1.5)	5 (4.3)	--
Healthy Weight [*n* (%)]	75 (46.8)	68 (51.1)	71 (61.2)	0.68 ^a^
Overweight [*n* (%)]	30 (18.7)	22 (16.5)	17 (14.6)	0.56 ^a^
Obesity [*n* (%)]	48 (30.0)	40 (30.8)	23 (19.8)	0.42 ^a^
Distance school-agricultural field (m)	431.3	388.1	2383.3	---

Values are presented as geometric means. 95% CI, 95% confidence interval. BMI: Body mass index, classified based on the U.S. Centers for Disease Control and Prevention [62] in underweight: <5th percentile; healthy weight: ≥5th to <85th percentile; overweight: ≥85th to <95th percentile; and obesity: ≥95th percentile. The data were analyzed by the Chi-square (^a^) and Kruskal–Wallis test (^b^). *p* < 0.05 was considered statistically significant.

**Table 2:** Urinary dialkylphosphate metabolites in children.

Metabolite (nmol/g Creat)	Community A		Community B		Community C		*p* Value	Min	Percentiles			Max
	GM	95% CI	GM	95% CI	GM	95% CI			p25	p50	p75	
DEP	10.2	7.2, 14.5	--	--	43.4	26.9, 70.1	<0.001	3.9	10.38	20.11	25.3	391.9
DETP	1977.8	1878.6, 2082.2	2195.8	2127.5, 2266.3	672.1	461.7, 978.3	<0.001	14.2	1334.8	2060.8	2228.1	2852.9
DEDTP	183.7	174.9, 193.0	--	--	--	--	--	161.7	161.7	161.7	210.5	210.5
DMP	37.3	37.3, 37.3	--	--	--	--	--	37.3	37.29	37.29	37.29	37.3
DMTP	370.6	364.0, 377.3	241.5	231.3, 252.2	276.3	260.4, 293.2	<0.001	187.2	226.6	280.1	368.0	438.4
DMDTP	553.9	528.8, 580.1	450.1	434.2, 466.7	188.2	169.6, 209.0	<0.001	105.0	373.6	418.5	537.4	727.0
ΣDEP	2059.5	1973.7, 2149.1	2195.8	2127.5, 2266.3	1195.3	1067.0, 1338.9	<0.001	406.1	1381.1	2060.8	2239.9	2852.9
ΣDMP	940.3	920.0, 961.1	693.4	669.0, 718.7	467.2	432.7, 504.4	<0.001	323.1	595.3	684.6	961.0	1059.1
ΣDAP	3019.8	2938.0, 3103.9	2892.5	2803.6, 2984.2	1501.0	1311.1, 1718.3	<0.001	406.1	2249.8	2823.3	2958.2	3859.2

GM: geometric mean; 95% CI: 95% confidence interval; Min: minimum; Max: maximum. The concentrations of DAPs were corrected by creatinine levels (μg/g creat). O,O-diethylphosphate (DEP), O,O-diethylthiophosphate (DETP), O,O-diethyldithiophosphate (DEDTP), O,O-dimethylphosphate (DMP), O,O-dimethylthiophosphate (DMTP), and O,O-dimethyldithiophosphate (DMDTP). DEP, DETP, DEDTP, DMP, DMTP, and DMDTP. ΣDEP: DEP + DETP + DEDTP. ΣDMP: DMP + DMTP + DMDTP. ΣDAP: DEP + DETP + DEDTP + DMP + DMTP + DMDTP. The *p* values were obtained by Kruskal–Wallis and post hoc Dunn’s test. *p* < 0.05 was considered statistically significant.

**Table 3:** Significant effect of exposed communities and pesticide exposure index on telomere length.

	Community	β	95% CI	PEI	β	95% CI
Unadjusted models	Community A	−117.1 ***	−182.9, −51.3	Moderate exposure	−83.1 *	−156.3, −9.9
	Community B	−131.3 ***	−199.1, −63.6	High exposure	−87.6 **	−152.0, −23.2
Adjusted models	Community A	−114.7 ***	−180.6, −48.8	Moderate exposure	−82.8 *	−156.6, −9.1
	Community B	−129.2 ***	−197.5, −60.9	High exposure	−85.8 **	−150.3, −21.3

Linear regression coefficients (β) and confidence intervals (CI) obtained from unadjusted and age- and sex-adjusted generalized linear models are shown. Linear regression evaluates the association of variables using Community C as reference. PEI: Pesticide exposure index. Statistical significance * *p* < 0.05, ** *p* < 0.01, *** *p* < 0.001.

## Data Availability

The original contributions presented in this study are included in the article. Further inquiries can be directed to the corresponding authors.
